# Analysis of umbilical cord tissue as an indicator of *in utero* exposure to toxic adulterating substances

**DOI:** 10.3389/fped.2023.1127020

**Published:** 2023-03-21

**Authors:** Kari M. Midthun, Brandon N. Nelson, Frederick G. Strathmann, Thom Browne, Barry K. Logan

**Affiliations:** ^1^ NMS Labs, Horsham, PA, United States; ^2^ MOBILion Systems, Inc., Chadds Ford, PA, United States; ^3^ Colombo Plan Secretariat, Colombo, Sri Lanka; ^4^ Center for Forensic Science Research and Education (CFSRE) at the Fredric Rieders Family Foundation, Willow Grove, PA, United States

**Keywords:** umbilical cord, adulterant, levamisole, dipyrone, phenacetin, xylazine, neonate

## Abstract

*In utero* drug exposure is a significant public health threat to the well-being and normal development of the neonate. Recently, testing of umbilical cord tissue (UCT) has been employed to measure illicit drug exposure, as drugs used by the mother during the third trimester may be retained in the UCT. Focus has also been given to potential adverse health effects among drug users, resulting from exposure to pharmacologically active adulterants and cutting agents in the street drug supply. The *in utero* effects of these substances have not been well studied in humans, nor has their presence been demonstrated as a means for assessing adverse health effects in the neonate. Here, we describe the application of a novel test method to analyze UCT for the presence of more than 20 common adulterating/cutting substances via LC/Q-TOF. In total, 300 de-identified UCT samples were analyzed–all had previously tested positive for cocaine or opiates. Generally, the positivity rates of individual compounds were similar between the Cocaine and Opiates Subgroups, apart from levamisole, xylazine, dipyrone (metabolites), and promethazine. Many of the adulterants used in the street drug supply do have legitimate medicinal/therapeutic uses, including several of the compounds most frequently detected in this study. Caffeine and lidocaine were the most frequently identified compounds both individually (>70% each) and in combination with each other. Alternatively, levamisole, an adulterant with no legitimate therapeutic use, was present in 12% of cases. Importantly, this data demonstrates that the detection of traditional drugs of abuse may serve as indicators of potential *in utero* exposure to toxic adulterating substances during gestation. While there is cause for concern with respect to any unintentional drug exposure, illicit drug use during pregnancy, including uncontrolled dosing, poly-adulterant consumption, and the interactions of these drug mixtures, produces a significant public health threat to the neonate which warrants further study.

## 1. Introduction

*In utero* drug exposure has been recognized as a significant public health threat to the well-being and normal development of the neonate (1, 2). Currently, 25 states and the District of Columbia require health care professionals to report suspected prenatal drug use (3). While reporting policies, consequences of reporting, and treatment options vary between states (4), 8 states require testing for prenatal drug exposure when drug use is suspected (3). Consequently, physicians frequently order monitoring and drug testing of pregnant women whose drug-use habits have been identified as putting the health of the mother and fetus at risk (5, 6). This monitoring may be done during pregnancy via urine and/or hair drug testing of the mother to assess recent or long-term drug exposure, respectively. Traditionally, monitoring has been performed following birth, by the collection and testing of meconium, for evidence of maternal drug exposure during the pregnancy. More recently, testing of umbilical cord tissue (UCT) to measure illicit drug exposure has been employed for this purpose. It has been demonstrated that drugs used by the mother during the third trimester may be retained in the cord tissue, and the detection of drugs in the tissue can be used to identify neonates for follow-up monitoring to assess potential health impacts (7), such as agitation, neonatal abstinence syndrome (NAS) (8) and drug withdrawal (9). Additionally, UCT offers several advantages over meconium as a toxicological sample, including immediate availability following birth, larger specimen volume, homogeneous composition, and ease of collection. In contrast, meconium may be released prematurely, before or during delivery, or excreted as a heterogeneous mixture over the first several days of the infant's life, making proper collection for testing purposes difficult (7, 10). Current drug testing practices for UCT have focused on the traditional drugs of abuse, including cocaine, heroin, and methamphetamine; substances with the greatest perceived risk for neonatal health effects.

Recently, additional attention has been given to the potential adverse health effects among drug users resulting from exposure to pharmacologically active adulterants and cutting agents in the street drug supply (11–13). Cutting agents are defined as bulking agents, or pharmacologically inactive diluents added to street drugs for the purpose of adding bulk to dilute the drug and increase the number of doses that can be sold from a given weight of active illicit drug. Adulterants are used for the same purpose but are differentiated in that they have pharmacological activity that may enhance, counter, or alter the illicit drug's effects or exert additional adverse effects on the drug user. The most common cutting agents are sugars and starch (14). Common adulterating substances include levamisole, aminopyrine, diltiazem, and phenacetin, as well as acetaminophen, caffeine, diphenhydramine, quinine, tramadol and xylazine (14, 15). Since the effects of these substances can be significant, and are typically ingested unknowingly by the drug user, they have been designated as Toxic Adulterants (11). Other emerging trends in the adulteration of the illicit drug supply include the adulteration of traditional street drugs with novel psychoactive substances (NPS) in the form of mixed powders and counterfeit and falsified pills, resulting in additional exposure and risk to the user (16). In recent events, adulteration of street drugs has been achieved by the addition of synthetic cannabinoids (17), fentanyl (18), and anticoagulants (19). A recent review considers the many known and potential adverse effects to the mother and child from exposure to drug adulteration from NPS (20).

The health effects of exposure to many of these substances on the fetus *in utero* have not been well studied in humans, with the exception of caffeine (21, 22) and acetaminophen (23, 24). Anecdotal reports of adverse effects resulting from maternal exposure to some substances have been identified, such as metamizole (dipyrone) (25) and ketamine (26). There are also concerning indications from animal studies of the potential for effects on gestation for toxic adulterating substances such as xylazine (27), tramadol (28), ketamine (29), and quetiapine (30). The persistence of toxic adulterating substances in umbilical cord tissue has likewise not been demonstrated as a potential means for assessing the corresponding adverse health effects on the neonate.

This report describes the application of a previously validated LC/Q-TOF method to analyze a large population of UCT samples for the presence of common toxic adulterating substances. Focus was given to adulterating and cutting agent compounds to better identify the scope and breadth of *in utero* drug exposure. The method was used to assess 300 UCT samples collected following delivery from drug-exposed mothers. The UCT samples tested positive for opiate or cocaine analytes prior to their use in this study.

## 2. Methods

### 2.1. Scope

Commonly identified adulterating and/or cutting agents from recent studies and analyses of seized drug exhibits (11, 12, 31, 32) were used as the basis for the scope of this analysis. Test analytes of interest include: acetaminophen, aminopyrine and metamizole (dipyrone) breakdown products (4-aminoantipyrine, 4-formylaminoantipyrine, 4-methylaminoantipyrine), benzocaine, caffeine, dextromethorphan, diltiazem, diphenhydramine, ketamine, lidocaine, levamisole, noxiptiline, phenacetin, procaine, promethazine, quetiapine, quinine, tramadol, and xylazine. Internal standards used were acetaminophen-D4, ketamine D-4, and promethazine-D3.

### 2.2. Sample preparation

De-identified human UCT samples were provided by United States Drug Testing Laboratories, Inc. (USDTL, Des Plaines, IL). Samples had previously tested positive for cocaine (i.e., benzoylecgonine) or opiate analytes (i.e., 6-monoacetylmorphine (6-MAM), morphine, and/or meconin). The full specimen tissue was rinsed of any external or residual blood with deionized water and patted dry. Approximately one gram was sampled, rinsed in saline solution, and patted dry again to avoid any contamination with residual blood. The sample was cut into small pieces and accurately weighed. A second aliquot was similarly prepared as the duplicate required for standard addition analysis.

### 2.3. Extraction

Extraction procedures utilizing a standard addition process have been described previously (33). Briefly, duplicate samples were prepared in plastic culture tubes. For each sample, one tube remained unaltered (e.g., blank sample) while the other tube was fortified with a mixture of all 21 analytes of interest (e.g., spiked sample). All tubes received an aliquot of internal standard solution to verify extraction efficacy. Three cleaned, stainless-steel screws were added to each tube, along with acetone/acetonitrile. Tissues were homogenized and then centrifuged. The supernatants were removed, dried, and reconstituted for extraction using solid phase extraction (SPE) techniques. The eluates were collected, evaporated to dryness, and reconstituted for injection. By using the self-controlling method of standard addition, no additional control samples were required for qualitative analysis.

### 2.4. Instrumental and data analysis

Analysis was performed using liquid chromatography tandem quadrupole time of flight mass spectrometry (LC/Q-TOF) as described previously (33). Samples were analyzed on an Agilent Technologies 1,290 Infinity UHPLC with AJS-ESI 6,545 QTOF with a run time of 48 s, using data dependent acquisition (DDA) for data collection.

Agilent MassHunter Qualitative Analysis 10.0 software was used for data analysis. Retention time (RT, minutes), database scores (DBS), and library search scores (LSS) were used to determine positivity for all drugs in the scope based on validated criteria established during method development (33). A sample was considered positive if it met or exceeded the established criteria for RT, DBS, and LSS. Any results that met or exceeded the LSS, but did not meet RT or DBS criteria were manually reviewed for positivity. Results which did not meet these criteria were reported as none detected.

## 3. Results

In total, 300 UCT samples were received from USDTL and analyzed using the method described previously (33). From this population, 183 (61%) samples were reported positive for cocaine, and 117 (39%) were positive for opiates (morphine, 6-MAM and/or meconin). All 117 opiate positive samples were positive for the presence of morphine. Of note, no samples provided were positive for both opiates and cocaine.

With the 300 samples tested, 293 UCT samples (>97%) were found positive for one or more of the adulterant analytes within the scope of testing. Table 1 lists the positivity rate for each adulterant analyte in the scope of the assay. Positivity rates were calculated as a percentage of the number of patients testing positive for that adulterating substance within the applicable test population: Total Cohort, Cocaine Subgroup, and/or Opiates Subgroup. Seven UCT samples were found to be none detected for the full scope of analytes—3 samples from the Cocaine Subgroup and 4 samples from the Opiates Subgroup.

**Table 1:** Positivity of adulterants as totals and percentages (%) of total numbers of patients in total cohort, cocaine subgroup, and opiates subgroup, respectively.

Compound	Total Toxic Adulterant Positives	Positives as % of Total Patients	Cocaine Toxic Adulterant Positives	Positives as % of Cocaine Patients	Opiates Toxic Adulterant Positives	Positives as % of Opiates Patients
Caffeine	228	76%	136	74%	92	79%
Lidocaine	216	72%	138	75%	78	67%
Diphenhydramine	81	27%	55	30%	26	22%
Quetiapine	73	24%	44	24%	29	25%
Levamisole	35	12%	32	18%	3	3%
Acetaminophen	33	11%	21	12%	12	10%
Quinine	30	10%	14	8%	16	14%
Tramadol	18	6%	8	4%	10	8%
Phenacetin	15	5%	9	5%	6	5%
Dextromethorphan	8	3%	5	3%	3	3%
Promethazine	6	2%	5	3%	1	0.9%
4-Methylaminoantipyrine	3	1%	0	0%	3	3%
Xylazine	3	1%	0	0%	3	3%
4-Formylaminoantipyrine	2	0.7%	1	0.5%	1	0.9%
Ketamine	2	0.7%	1	0.5%	1	0.9%
Aminopyrine	1	0.3%	0	0%	1	0.9%
4-Aminoantipyrine	1	0.3%	0	0%	1	0.9%
Procaine	1	0.3%	0	0%	1	0.9%
Noxiptiline	1	0.3%	1	0.5%	0	0%
Benzocaine	0	0%	0	0%	0	0%
Diltiazem	0	0%	0	0%	0	0%
Total (Patients)	300		183		117	

**Figure 1:**
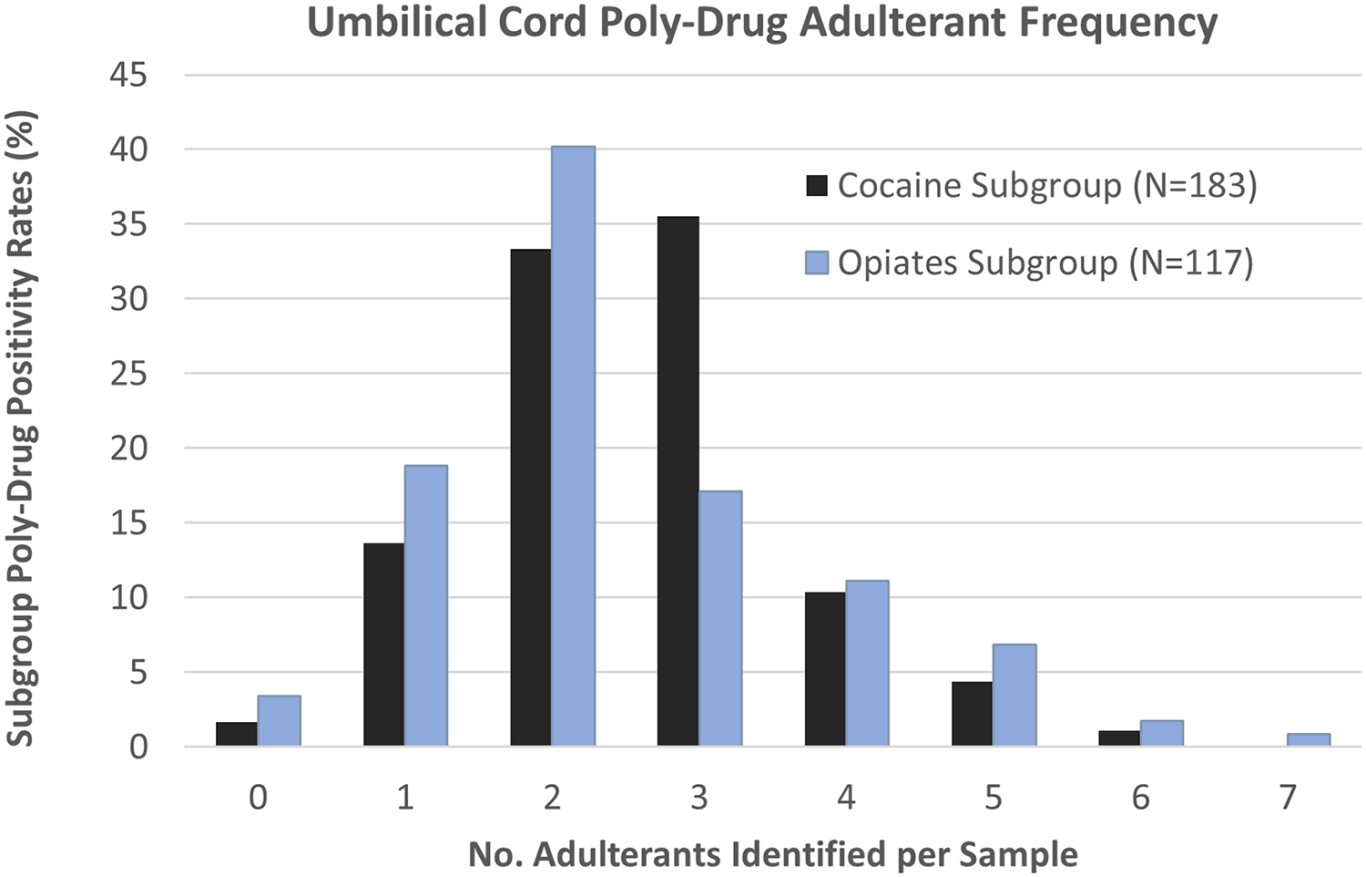
Umbilical cord tissue poly-drug adulterant frequency in Cocaine (black) and Opiates (blue) Subgroups. Individual samples were found to contain anywhere from 0 to 7 adulterating substances.

Results were analyzed to determine the frequency of poly-substance cases (Figure 1), with the overall presence of 2 adulterating substances being the most common poly-drug combination seen (*n* = 108, 36%) by the Total Cohort. The Cocaine Subgroup saw poly-drug combinations ranging from 0 to 6 different adulterating substances. However, more than 80% (*n* = 151) of the Cocaine Subgroup contained between 1 and 3 adulterants, with the presence of 2 and/or 3 adulterating substances representing more than two-thirds of the cocaine positive samples. The Opiates Subgroup contained between 0 and 7 different adulterating substances with the presence of 2 drugs being the most common combination (*n* = 47, 40%). Combinations of 4 or more adulterants were more commonly seen in the Opiates Subgroup (21%) than the Cocaine Subgroup (16%).

**Figure 2:**
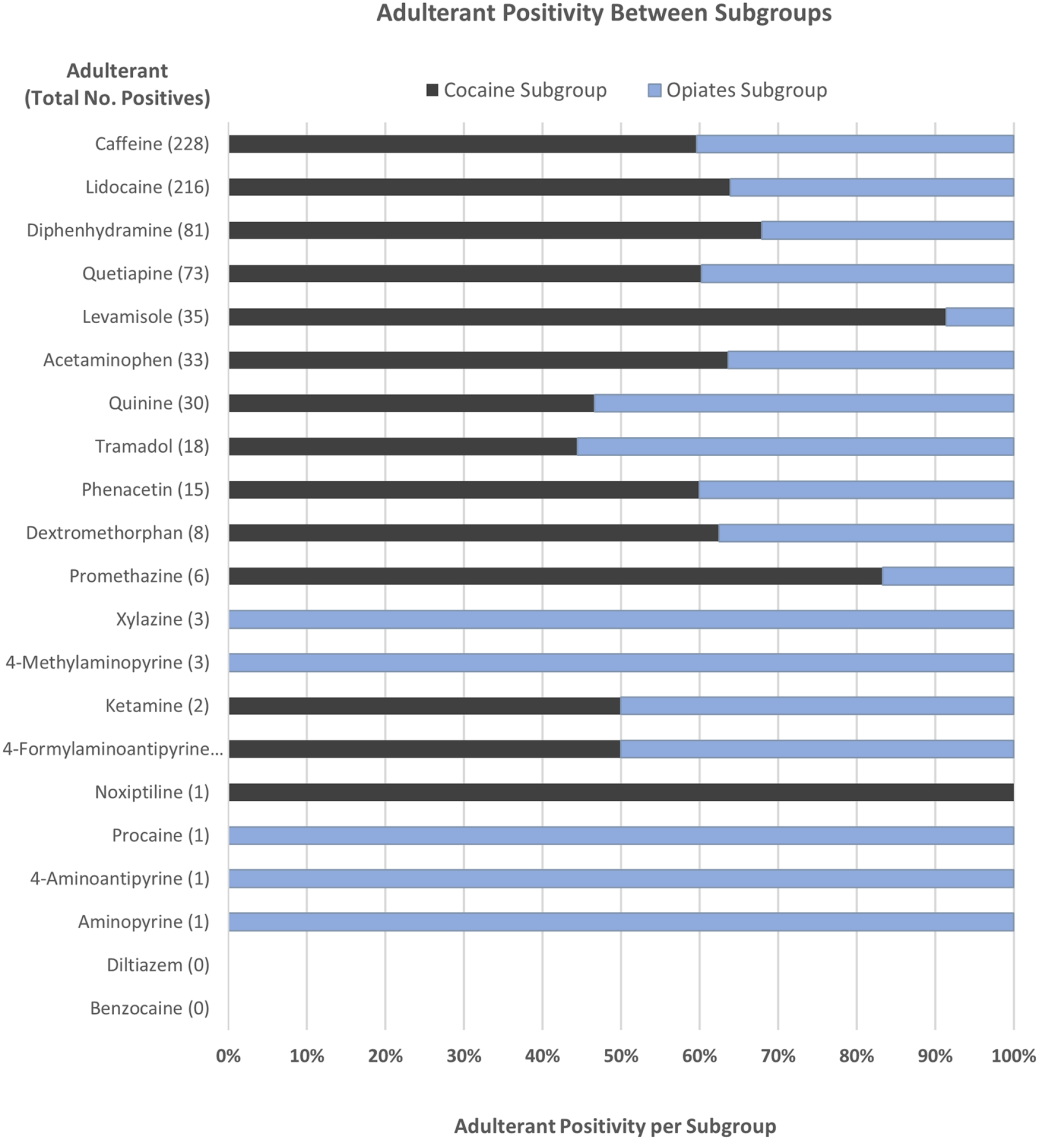
Comparison of toxic adulterant positivity between the Cocaine (black) and Opiates (blue) Subgroups. Positivity rates were determined as a percentage of the total number of positive cases for each adulterant.

The positivity rate for each analyte was also compared between the two subgroups to determine whether the presence of an analyte was more commonly associated with one drug subgroup over the other as shown in Figure 2. The graph demonstrates these occurrences with several of the adulterating substances only being found in the Opiates Subgroup population. These include xylazine, 4-methylaminopyrine, procaine, 4-aminoantipyrine, and aminopyrine. Conversely, noxiptiline, levamisole, promethazine, and diphenhydramine were more predominant (>67%) in the Cocaine Subgroup, suggesting that cocaine may be more commonly adulterated with these substances versus opiate formulations. It is also important to point out that the larger population of cocaine positive samples used in this data set may skew some analyte data towards the Cocaine Subgroup. Among these, the positivity rates of caffeine, lidocaine, quetiapine, acetaminophen, phenacetin, and dextromethorphan are higher in the Cocaine Subgroup, though it is unclear if this is a trend from the data or due to the larger cocaine positive population surveyed. Alternately, quinine and tramadol are slightly associated with the Opiates Subgroup.

**Figure 3:**
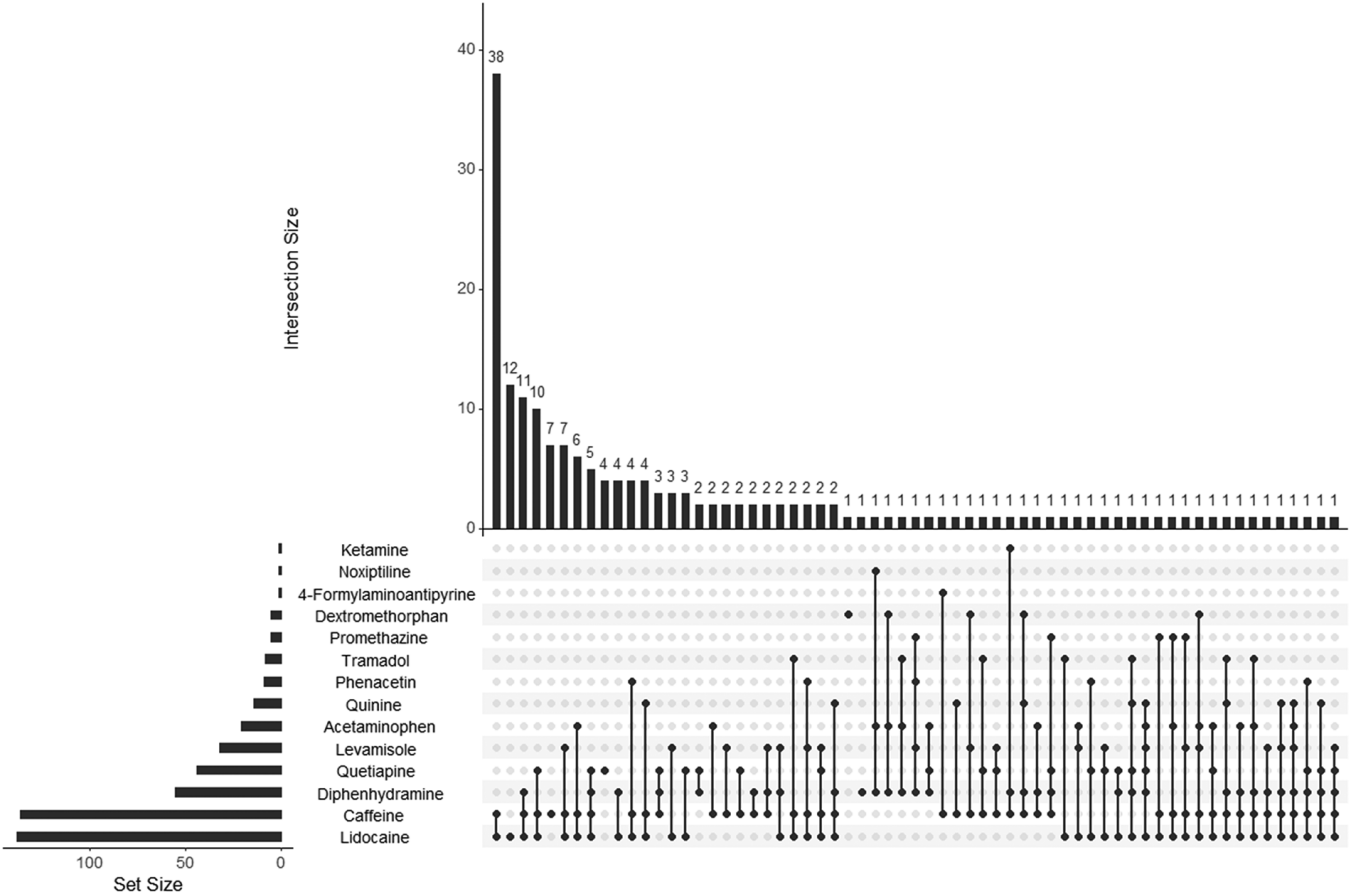
UpSet plot showing adulterant frequency (set size) and poly-drug intersections in umbilical cord tissue from the Cocaine Subgroup. The set size bar graph indicates the total number of positive cases identified for that individual adulterant within the Cocaine Subgroup. Poly-drug combinations, or intersections, are illustrated by the dot diagrams with connecting lines indicating adulterants found in combination with one another. A lone dot indicates a single drug finding. The frequency of each intersection is shown by the histogram at the top of the plot with the frequency number indicated for each combination seen. For example, the Cocaine Subgroup contained 138 lidocaine positive cases, 12 of which were positive for lidocaine-only. The following scope adulterants were not identified in any Cocaine Subgroup samples tested and were not included in the plot: 4-methylaminoantipyrine, xylazine, aminopyrine, 4-aminoantipyrine, procaine, benzocaine, and diltiazem.

**Figure 4:**
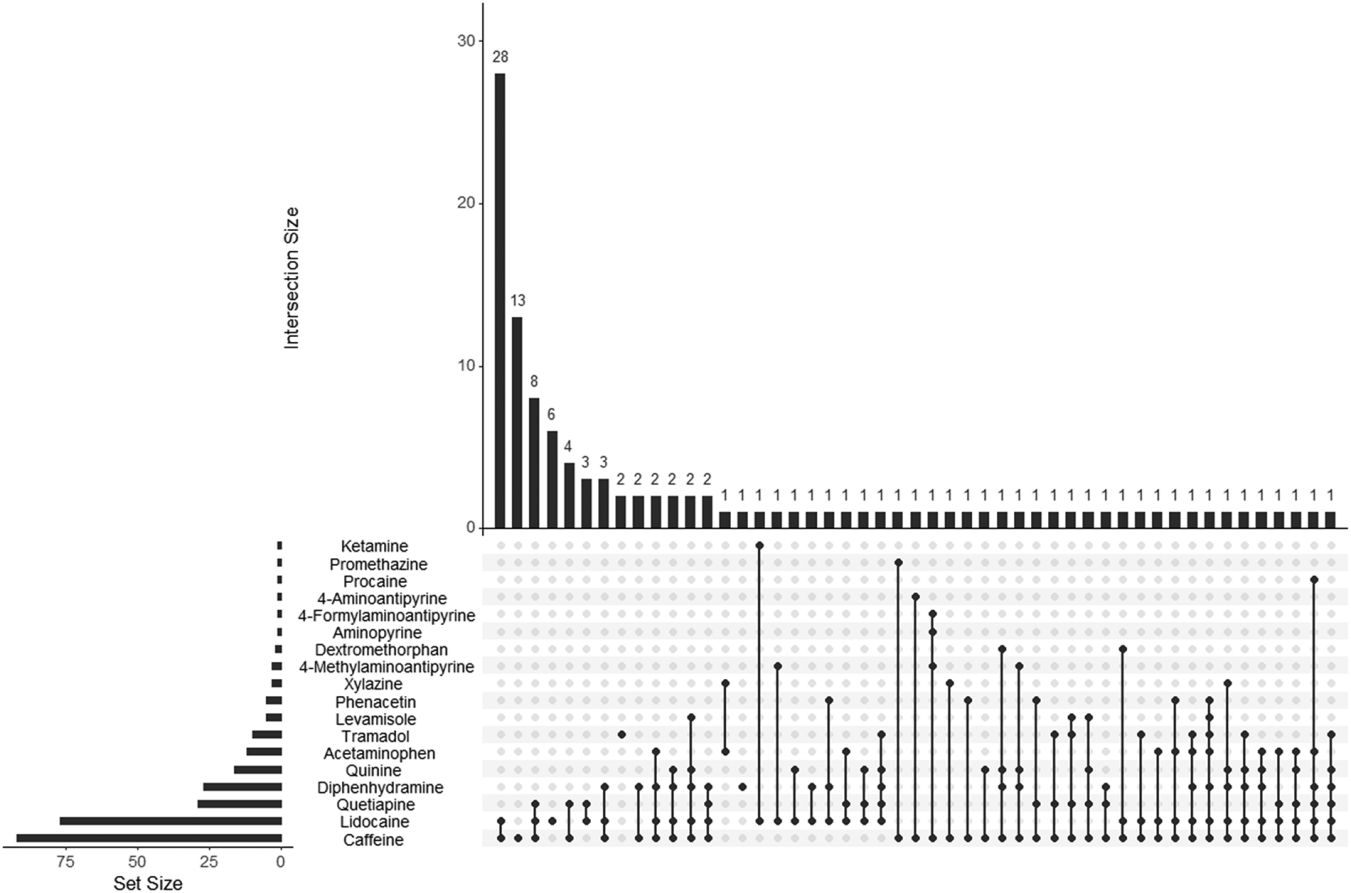
UpSet plot showing adulterant frequency (set size) and poly-drug intersections in umbilical cord tissue from the Opiates Subgroup. The set size bar graph indicates the total number of positive cases identified for that individual adulterant within the Opiates Subgroup. Poly-drug combinations, or intersections, are illustrated by the dot diagrams with connecting lines indicating adulterants found in combination with one another. A lone dot indicates a single drug finding. The frequency of each intersection is shown by the histogram at the top of the plot with the frequency number indicated for each combination seen. For example, the Opiates Subgroup contained 92 caffeine positive cases, 28 of which were positive for the combination of caffeine and lidocaine. The following scope adulterants were not identified in any Opiates Subgroup samples tested and were not included in the plot: noxiptiline, benzocaine, and diltiazem.

The subgroups were analyzed separately to determine the most common adulterant combinations encountered (Figures 3, 4). Overall, the combination of caffeine and lidocaine was the most encountered finding for both the Cocaine Subgroup (*n* = 38, 20%) and the Opiates Subgroup (*n* = 28, 23%). Figure 3 illustrates that the next most common Cocaine Subgroup finding was lidocaine-only (*n* = 12, 6.5%) followed by combinations of caffeine and lidocaine with diphenhydramine (*n* = 11, 6.0%), quetiapine (*n* = 10, 5.4%), and levamisole (*n* = 7, 3.8%), respectively. The presence of caffeine-only was seen in 3.8% (*n* = 7) of the Cocaine Subgroup. In contrast to this, caffeine-only was the second most common finding (*n* = 13, 11%) for the Opiates Subgroup (Figure 4). As shown by Figure 4, caffeine-only was followed by the triplet combination of caffeine, lidocaine, and quetiapine (*n* = 8, 6.8%); lidocaine-only (*n* = 6, 5.1%); and combinations of quetiapine with either caffeine (*n* = 4, 3.4%) or lidocaine (*n* = 3, 2.5%).

## 4. Discussion

Many of the substances known to be used as toxic adulterants in the street drug supply also have legitimate legal or therapeutic uses, including several of the compounds most frequently detected in this study. However, little is known about the impacts and adverse effects for many of these compounds on the fetus/neonate, particularly when consumption exceeds dietary and therapeutic recommendations. Unlike other specimens in the various fields of toxicology, UCT drug testing does not currently have established concentration ranges or cutoff levels. As such, results from this study are reported qualitatively and it is not possible to draw direct conclusions about any toxic effects experienced by the neonates in this study. This would require further investigation with careful monitoring of the neonate's symptoms and development, as well as establishing a non-drug exposed maternal control group for comparison.

### 4.1. Adulterants whose origins cannot be attributed specifically to illicit drug use

Caffeine may be consumed via a wide variety of commercial products, including soft drinks, mixers, coffee, tea, energy drinks and chocolate, or taken therapeutically to treat maladies including headaches and drowsiness. Lidocaine is a common anesthetic administered during medical procedures, including childbirth. Quinine can be found as the primary flavoring ingredient in tonic water and as a common prescription treatment for malaria. Due to their presence in commercial products and routine therapeutic uses, presence of these compounds in a UCT sample cannot be definitively attributed to adulterated street drug consumption. Additionally, common adulterating agents, such as acetaminophen, dextromethorphan, and diphenhydramine are available as conventional over-the-counter (OTC) analgesics, antitussives, and decongestants/sleep aids, respectively. In terms of combinations of adulterants, the most common overall combination involving caffeine and lidocaine (*n* = 66, 22%) cannot be attributed definitively to illicit drug use.

While these non-prescription drugs are safe in moderation, the doses in illicit street drug preparations are uncontrolled and unknown to the user, putting them at risk of adverse effects. Equally concerning are studies which have shown that excessive consumption of more ubiquitous compounds during pregnancy, such as caffeine and acetaminophen, may increase risks of both fetal growth restrictions and developmental issues (21, 34–36). From this study, the data clearly shows that these compounds are incorporated into the UCT, providing direct exposure to the neonate. What remains unclear is what impact these uncontrolled and unknown doses may have on the neonate both short term and long-term.

Consideration must also be given to drugs that are prescribed as part of routine dental and medical care. For example, lidocaine is administered locally during dental procedures and also during childbirth for pain relief, nerve blocking, and for treating peritoneal stretching and episiotomies (37, 38). Similarly, fentanyl is a common operative analgesic that can also be found routinely in epidurals for pain relief during labor and delivery (39). Ketamine (40), procaine (41), and benzocaine (42) can be administered as anesthetics during medical and dental procedures, and tramadol may be used as a prescription treatment for moderate to severe pain (43). Noxiptiline, a tricyclic antidepressant developed in the 1970s in Europe (44), is not approved or marketed in the United States (45). Complicating things even further, several of the adulterating agents can be prescribed for the treatment of chronic conditions, including the antipsychotic quetiapine, the calcium channel blocker diltiazem, and the multi-purpose drug promethazine. Thus, the presence of an adulterating compound in UCT, alone or in combination, is unable to be fully interpreted without consideration of prenatal and perinatal care. In this study, cases were de-identified as to the donor, such that no clinical histories on the patients were available to differentiate illicit exposure from legitimate routine, therapeutic, or dietary sources of these substances.

Quinine was present in 10% of all cases (*n* = 30), and in 8% and 14% of the Cocaine and Opiates Subgroups, respectively. While present in the diet as a component of drink mixers, and used routinely in the treatment of malaria, quinine is also commonly used to cut or adulterate cocaine and heroin (11, 12), and this is reflected in the data. When combined with heroin, this substance mimics the “rush” by the hypotensive effect and the bitter taste is similar to that of heroin (46). Quinine can result in cardiovascular toxicity including abnormal heart rhythms and hemolysis (47), or produce other adverse effects if taken in excess. For example, high doses of quinine have been used in some countries in attempts to induce abortions. This resulted in a number of adverse effects for the mothers and neonates in cases where the drug failed to induce an abortion, including blindness, deafness, hemoglobinuria, reversible renal failure, and maternal death (48).

Diphenhydramine is generally considered safe to consume during pregnancy when taken therapeutically to treat symptoms such as allergic rhinitis, headache, insomnia, and postpartum depression (49, 50). However, these studies did not assess outcomes for potentially high doses of diphenhydramine, as might be observed from chronic administration of adulterated cocaine or heroin. Of note, diphenhydramine has been cited in the literature as having potential risks to the health of the mother and/or fetus (51–53). Hyperemesis gravidarum (HG), an extreme persistent nausea during pregnancy, is frequently treated with chronic antihistamine administration. One report indicates that poor neonatal outcomes were significantly greater in women with this condition and were positively associated with gestational hypertension, early start of HG symptoms, and antihistamine use (54). While a few studies have suggested the risk of birth defects resulting from antihistamine use during the first trimester, the results have been inconsistent and there is an overall lack of strong evidence to conclude that any birth defects are associated with therapeutic antihistamine exposure during early pregnancy (55–57). Other authors have called for more studies of OTC medications during pregnancy (52, 58).

Quetiapine (Seroquel®) is a potent second-generation antipsychotic drug used to treat schizophrenia in adults and children. It can be subject to abuse in its own right (59), and has recently been identified as a potential toxic adulterating agent due to having been detected in 10 seized drug exhibits in Kentucky and Vermont (12). Quetiapine was found in UCT in 73 (24%) of the cases in this study, although it cannot be determined if this was from prescription use, from adulteration of street drugs, or a combination of both. In studies regarding women prescribed quetiapine during their first trimester of pregnancy, the authors found no increased risk for major congenital malformations in the infant (60, 61). Case reports identify numerous examples of children (62, 63), adults (64, 65), and a pregnant mother (66) surviving acute quetiapine poisonings—their most common symptoms being tachycardia, QT prolongation, and loss of consciousness. Quetiapine-associated overdose deaths have also been reported (65, 67, 68).

### 4.2. Adulterants of illicit origin and their effects

Levamisole, phenacetin, metamizole (dipyrone), and xylazine are of interest in this patient study population as they are not approved in the United States for therapeutic use in humans. All four of these drugs were detected in the UCT from this study, representing likely exposure of the mother and the fetus to these toxic adulterants at unknown doses through the illicit drug supply. Importantly, this is the first documentation of the incorporation of these substances into the cord tissue and, consequently, of transfer to the developing fetal tissue.

Levamisole was found predominantly in the Total Cohort in 12% of cases (*n* = 35); and in cases positive for cocaine at 18% (*n* = 32), but in only 3% (*n* = 3) of the opiate positive cases. These findings are consistent with previously described drug seizure reports, such that levamisole is more commonly seen as a cocaine adulterant but can also be added to fentanyl and heroin supplies (11, 12). Levamisole is an antihelminthic agent used in veterinary medicine but has also been used illicitly as a cocaine adulterant since 2002 (69, 70). This timing coincides with levamisole's withdrawal from United States and Canadian pharmaceutical markets due to reports of serious adverse effects (71). The concentration of levamisole in the domestic United States cocaine supply has steadily increased since it was first detected (72). After acute intake, nausea, diarrhea, and dizziness are common effects of this drug. After prolonged intake, muscle pain, headache, fever, insomnia, dizziness, and convulsions can occur. Potential complications associated with use of levamisole-laced cocaine include neutropenia, agranulocytosis, arthralgias, methemoglobinemia, purpura retiform, systemic vasculitis, cutaneous necrosis, intravascular thrombosis, and skin necrosis (73, 74). To the authors' knowledge, the potential adverse effects of this drug to the developing fetus, at doses associated with typical recreational cocaine use, have not been evaluated. Prior studies have shown that doses of levamisole ingested by drug users are within the toxic range, raising the risk of adverse outcomes for both the mother and the fetus (75).

Phenacetin was detected in 5% (*n* = 15) of the Total Cohort, representing 5% of the Cocaine Subgroup (*n* = 9) and 5% of the Opiates Subgroup (*n* = 6), respectively. Phenacetin is a pain-relieving and antipyretic drug, which can metabolize into acetaminophen. Phenacetin was banned in the United States in 1983 by the FDA (76) due to concerns raised about carcinogenesis, nephropathy, and hemolytic anemia in children (77, 78). In more recent years, phenacetin has been observed as a common cutting agent in street drugs (11, 12), especially as a cocaine additive (79–82). Phenacetin's carcinogenicity has been attributed to the metabolic bioactivation of several reactive downstream metabolites and intermediates, all with variable toxicological consequences (83).

Metamizole/dipyrone and aminopyrine have had similar issues to phenacetin as far as pharmaceutical usage, ranging from removal from the United States market in the 1930s (aminopyrine) (84) to being banned by the FDA in 1977 (dipyrone) and 1979 (metamizole) (76). While proven analgesics and antipyretics, both metamizole/dipyrone and aminopyrine were found to cause severe and sometimes fatal agranulocytosis, leading to their removal from markets in many countries (84–87). The compounds also share a common metabolic pathway. Once inside the body, they quickly metabolize to active 4-methylaminoantipyrine, which can be further broken down into a number of different compounds, including active 4-aminoantipyrine and inactive 4-formylaminoantipyrine (88). Due to rapid metabolism of metamizole/dipyrone, attention was focused on detecting the presence of the metabolites and aminopyrine for the purposes of this study. Interestingly, the metabolites were detected in the Total Cohort, but with widely varying results. The primary metabolite, 4-methylaminoantipyrine, was identified in 3 total cases (1%), all of which came from the Opiates Subgroup. The inactive metabolite, 4-formylaminoantipyrine, was detected in 2 total cases (0.7%), one coming from the Cocaine Subgroup and one from Opiates. And finally, active 4-aminoantipyrine was detected in a single case from the Opiates Subgroup—this was the only case found to contain all three metabolites. The single occurrence of aminopyrine also came from a case in the Opiates Subgroup; however, no other metabolites were present concomitantly. In a systematic review of literature regarding the use of metamizole/dipyrone as a pediatric analgesic, the authors could not conclusively determine the risk of agranulocytosis in children due to the limited amount of data available (89). They also could not find evidence to support that metamizole/dipyrone was superior to other non-steroidal anti-inflammatory drugs (NSAIDs) still on the market (87, 89). In general, the drug is not recommended for use with children (87); or if used, treatment duration should be kept as short as possible to minimize any possible adverse reactions (90). No similar reviews or studies could be located for aminopyrine. In one recent case report from Turkey, a 4-year-old boy developed life-threatening agranulocytosis and anemia after receiving dipyrone as treatment for a fever (91).

Xylazine, a veterinary analgesic not approved for human use, was found in 3 cases (1%), all of which were positive for opiates. Xylazine exposure in humans has been associated with hypotension, bradycardia, orthostatic hypotension, and respiratory depression, and with abscess, ulcerations, and other skin lesions in intravenous drug users (92–94). In more recent years, xylazine has emerged as a common drug adulterant (11, 12, 93), often present with heroin, fentanyl, and/or cocaine. At this time, very little is known about xylazine's pharmacokinetics or mechanism of action in humans. However, as an adulterant in heroin, cocaine, and fentanyl, it can potentiate sedation and respiratory depression, increasing risk of overdose (95). Xylazine does not respond to opioid reversal agents such as naloxone, nor is there a known pharmaceutical antidote specific to xylazine (96). What remains clear is that xylazine may be harmful to humans, particularly when taken concomitantly with other drugs which may further potentiate the effects and lead to toxic and/or fatal results (93).

With regards to illicit adulterants, levamisole in combination with caffeine and lidocaine was the most prevalent overall combination (*n* = 7, 3.8%), all of which were found in the Cocaine Subgroup. This was followed by a combination of phenacetin, caffeine, and lidocaine (*n* = 4, 2.2%), again only seen by the Cocaine Subgroup. Inasmuch as levamisole was the most common adulterant present in the Cocaine Subgroup (*n* = 22), there was no consistent pattern in terms of what other drugs it was found concomitant. Similarly, while the Opiates Subgroup predominated with the presence of xylazine and metamizole/dipyrone metabolites, there were no clear patterns of illicit drug combinations seen with this subgroup. Drug metabolism is different in children and adolescents, and illicit drugs and toxic adulterants can cause greater harm even at lower doses in the juvenile and adolescent brains (97, 98). Drugs and adulterants that suppress the immune system (i.e., deplete white blood cells) are an especially significant threat to children as their immune systems are not fully developed (99).

## 5. Conclusions

These data clearly demonstrate that substances other than traditional drugs of abuse can be detected in UCT. Additionally, these substances can act as markers for *in utero* exposure to potentially toxic adulterating substances during gestation where the umbilical cord also tested positive for cocaine or morphine (from heroin). While there is cause for concern with respect to illicit drug exposure and/or unintentional drug exposure at any age, the neonate population remains particularly vulnerable, as impacts and adverse effects are not well understood and may not be readily apparent at birth. Drug use during pregnancy is a recognized significant public health threat to the neonate with illicit drug use being the primary concern. As highlighted by this study, therapeutic, dietary, and illicit compounds can all be identified in combination in UCT through a high-risk neonatal population. Further studies are needed to better understand how uncontrolled dosing of these potentially toxic adulterating substances, the mixing of multiple adulterating substances, and the interaction of adulterants with each other and with illicit drugs of abuse may impact the health and development of the neonate. Thus, the gathering of neonatal outcome data, in terms of adverse events during delivery, neonatal toxicology results, and neonatal development indicators, should be collected to establish the significance of this newly documented exposure.

## Data Availability

The original contributions presented in the study are included in the article, further inquiries can be directed to the corresponding author.
